# Indirect Sensing of Subclinical Intramammary Infections in Dairy Herds with a Milking Robot

**DOI:** 10.3390/s23229036

**Published:** 2023-11-08

**Authors:** Ivars Lusis, Vita Antane, Andres Waldmann

**Affiliations:** 1 Faculty of Veterinary Medicine, Latvia University of Life Sciences and Technologies, Helmana 8, LV-3004 Jelgava, Latvia; vita.antane@lbtu.lv (V.A.);; 2 Institute of Veterinary Medicine and Animal Sciences, Estonian University of Life Sciences, Kreutzwaldi 62, 51006 Tartu, Estonia

**Keywords:** milk sensors, somatic cell count, intramammary infection, subclinical mastitis, minor mastitis pathogen, major mastitis pathogen, mastitis detection index

## Abstract

This study determined the impact of subclinical intramammary infections (IMIs), such as the major and minor udder pathogens (MaPs and MiPs), on the somatic cell count (SCC) in cow milk and investigated the possibilities of indirect sensing of the udder pathogens using the mastitis detection index (MDi) (DeLaval, Tumba, Sweden). The MDi incorporates quarter-level milk electrical conductivity, blood in milk, and milking interval. The case group (n = 21; MDi ≥ 1.4) was compared with the control group (n = 24; MDi < 1.4) for the presence of IMIs. The microbiological investigation of udder quarter foremilk samples was performed two times with an interval of 10 to 14 days. The case and control groups differed in terms of the occurrence of MaPs and MiPs in milk. During the continuous subclinical IMI and the episodic MaP infection, a higher SCC was detected compared with the episodic MiP infection or quarters without IMI. The novel finding of this study was that by using the milk quality sensor for the sensing of subclinical IMIs, there was an indication for the successful detection of episodic MaPs. However, the sensing of the continuous subclinical IMIs was not possible in the current study and still needs to be investigated.

## 1. Introduction

Usually, to follow up on subclinical mastitis caused by intramammary infections (IMIs) in a dairy herd, the cows with the highest somatic cell counts (SCCs) in their milk are selected for laboratory testing for pathogens in their milk. A more problematic approach would be to follow up the IMI based on the assumption that an elevated SCC > 200,000 cells/mL is indirect evidence of bacterial infection [1,2,3]. However, it would be a misleading assumption that cows with an SCC of less than 200,000 cells/mL can be considered free of an IMI. Even cows with SCCs below 100,000 cells/mL could have already shed pathogens from some of the quarters, such as *Staphylococcus aureus*, *Streptococcus uberis*, coagulase-negative staphylococci [4,5], or at least staphylococci and corynebacteria [6]. Operational characteristics such as diagnostic sensitivity and specificity to differentiate infected and non-infected cows correctly vary depending on the chosen SCC cut-off value. Using an SCC threshold lower than 200,000 cells/mL will result in labeling too many uninfected cows as infected [7,8]. From the abovementioned statements, it is evident that the SCC of a cow’s composite milk samples does not have enough diagnostic sensitivity to be useful in detecting subclinical IMIs in a dairy herd.

A more precise way to detect for IMIs is to check for an inflammatory response at the quarter level, characterized by an increase in the SCC in the foremilk from separate quarters of the udder. For scientific purposes, in line with a gold standard, it is recommended to take and test quarter-level samples weekly to show the same pathogen in at least two of three samples [9]. In many situations, it may be difficult to justify the additional expense of triplicate sampling compared with single sampling [10]. As described by Andersen et al., the Mastitis Research Workers’ Conference (2008) experts have proposed that the consensus pseudo-gold-standard rules for the classification of an udder quarter as IMI positive should be simplified to the following two rules: (1) the organism of interest is isolated on the test day at a concentration of 1000 colony-forming units per mL or more or (2) the organism of interest is isolated at least twice out of three consecutive weekly tests [11]. This principle confirms the continuation of the present bacterial infection after another week or two. The same approach can be applied to the non-infection status. However, some authors [12] suggest measuring the SCC at the quarter level and only those with SCCs below 100,000 cells/mL and free of pathogens may be recognized as non-infected. All other quarters that are free of pathogens but show an SCC higher than 100,000 cells/mL in milk should be categorized as infected [12].

For practical purposes, repeated milk sampling according to the pseudo-gold-standard is a difficult way to follow up and track the IMI in conventional milking, where a milking operator approaches each cow at every milking session. It is even more complicated in milking robots (MRs), which are automatic milking systems where milking is performed without the direct participation of an operator [13]. An MR is typically not designed to allow easy and safe access to the cow’s teats and udder foremilk sample collection [14]. This aspect is a distinct and essential difference between milk harvesting practices in MR compared with conventional milking. Thus, during the last decades, many sensors detecting indirect markers of IMI and milk quality changes [15] have been elaborated and applied in practical use in MRs to attract the attention of system users.

In the literature concerning bovine udder health issues, many authors [16,17,18,19,20,21,22,23,24,25,26,27] use a fundamental principle to classify mastitis pathogens into general groups—major pathogens (MaPs) and minor pathogens (MiPs). In addition, some authors use another group as a subcategory, “other major pathogens” [19].

MaPs, such as *Staphylococcus aureus*, *Streptococcus agalactiae*, *Streptococcus dysgalactiae*, *Streptococcus uberis*, and coliforms, mainly cause a strong inflammatory response and clinical illness and have a negative effect on future udder health and milk production [16]. MiPs, such as coagulase-negative staphylococci (CNS) and *Corynebacterium bovis*, cause mastitis but this mostly remains subclinical or shows only mild clinical signs. Continued infection with CNS leads to an increased milk SCC that affects udder health and milk quality and may be related to decreased milk production [16,21,22].

As referred to in earlier research [28,29], according to the software “Cow Monitoring in VMS” (VMSClient Version 2009 8.30.04.01, DeLaval International AB, Tumba, Sweden), the cow-level mastitis detection index (MDi), which takes into consideration two quarter-level parameters, namely the electrical conductivity of milk and the blood presence in milk, and the milking interval, gives an indication of the likelihood of mastitis in a cow. However, previous studies on the effectiveness of MDi data and a comparison with SCCs suggests an open question about the exact MDi thresholds for detecting clinical mastitis and diverting the abnormal milk [29,30].

From the practical point of view, any alarm generated from elevated SCCs or other parameters of udder health sensing (electrical conductivity, MDi) should be separated into two different tracks: perform a bacteriological testing of the milk sample or wait for the next alarm in further milking sessions. Because there is a high chance of becoming cured spontaneously, cows with slightly elevated SCCs may be left without immediate bacteriological testing [31]. This question demonstrates the dilemma between the high costs of bacteriological investigation (diagnostic work) if it is performed too often and the high costs of mastitis-caused economic losses if the infection is left untreated (therapeutic work). Some mastitis indicators, for example, have fluctuations and come back to normal levels spontaneously [29,32].

The current study aimed to estimate the impact of subclinical intramammary infections with the major and minor udder pathogens on the somatic cell count in milk and to investigate the possibilities of the indirect sensing of several bacterial pathogens in cow’s milk using milk quality detection sensors built-in to the milking robot (DeLaval, Tumba, Sweden). The specific task was to evaluate the presence continuity of the bacterial pathogens in milk depending on the mastitis detection index (MDi) value. According to the manufacturer’s information [32], the mastitis detection index helps to better manage milk’s somatic cell count in cows milked with robots. However, in this study we will determine its applicability for the sensing of mastitis pathogens in the milk. Previously, no study has tried to use the MDi for the indirect sensing of mastitis pathogens.

## 2. Materials and Methods

### 2.1. Herds and Cow Selection

Cows were selected from two dairy farms in Latvia. Before the current research was performed the farms had been equipped with an MR (VMS, DeLaval, Tumba, Sweden) for at least one year. Both farms used an MR only for a part of the herd; the rest of the cows were milked in a milking parlor. The first, a commercial dairy farm (Herd 1), managed a dairy herd of 135 cows, and on average, 60 cows were milked using an MR. The second, a university training and research farm (Herd 2), managed a dairy herd of 597 cows, and on average, 120 cows were milked using two MRs. For Herd 1, sand and chopped straw were used as bedding materials in free stalls. For Herd 2, walkways were covered by a rubber mat and free stalls by a rubber mattress. The MRs performed udder preparation for milking in both herds by an automatic cleaning procedure of teats combined with the stripping of foremilk. After the milking, the lower part of the udder was automatically sprayed with an iodine-based disinfecting solution (Prima Plus, DeLaval, Tumba, Sweden).

The study was carried out in October 2009 (Herd 1), October 2010 (Herd 1), and October 2011 (Herd 2). At the start of the study, the authors had no previous data (variability, effect size) for sample size calculations because this was the first attempt to explore the link between the MDi and subclinical IMIs. We carried out our study over three years. At the beginning, the study was performed in one herd; to test for external validity, more cases and controls were added from another herd. Cows were selected (Table 1) according to the mastitis detection index (MDi) [33]. As referred to in earlier research [28,29], MDi values of 1.4 or higher according to the software “CowMonitoring in VMS” (DeLaval International AB, Tumba, Sweden) indicate the likelihood of mastitis in cows. For comparison, a set of automatically collected data were obtained from the MR sensors during every milking session, and concurrent milk recording data were obtained from the cow pedigree cards of the Agricultural Data Center of Latvia.

The udder health alarm case group (n = 21) was composed of cows whose MDi values were equal to or greater than 1.4, which was visualized on the display of the herd management system. The control group (n = 24) was randomly selected from the other cows with MDi values of less than 1.4, forming matching pairs with animals of the case group by parity (1st, 2nd, 3rd, and greater) and by lactation phase (early 10–100, middle 101–200, late 201–305, and >305 days in milk). The distribution of cows according to the parity and lactation phase is shown in Table 1. None of the cows had an MDi alarm in the early lactation phase, so pairs of cows for this phase could not be created. However, the control group was supplemented with three other cows picked randomly from the early lactation cows with MDi values ranging from 1.0 to 1.3. The study was carried out in October, and only some cows were in an early lactation phase at this time.

### 2.2. Milk Sampling

Collection of the udder quarter foremilk samples for the microbiological investigation was performed aseptically two times with an interval of 10 to 14 days. At the time of milking, after the automatic teat cleaning process by the MR was completed, the MR was paused and manual disinfection of the teat ends with a cotton swab soaked in 70% ethanol was conducted. Immediately afterward, the milk sample was obtained manually from the teat. The samples were transported to the laboratory on ice in a thermo box within 12 h. Concurrently, foremilk samples from each udder quarter were collected and submitted to an accredited dairy laboratory to analyze the fat, protein, lactose, and SCC (SIA Piensaimnieka laboratorija, Ulbroka, Latvia).

### 2.3. Microbiological Testing

The milk samples were cultured following the methodology described in “*The Laboratory and Field Handbook on Bovine Mastitis*” by the National Mastitis Council [34,35]. A disposable loop (10 L^−6^) was used for the primary plating of the milk sample. Inoculated plates were incubated and checked 24 and 48 h later. The bacteria from the grown colonies were stained and evaluated according to Gram staining (Benex Limited, Dublin, Ireland), checked for catalase and oxidase production, and further subcultured onto differential media. The identification of mastitis pathogens to the group, genus, or species levels was performed on mannitol salt agar (Oxoid Limited, Altrincham, UK), bile esculin agar (Benex Limited, Dublin, Ireland), and MacConkey agar N2 (Biolife, Monza, Italy). The confirmatory test for *Staphylococcus aureus* (coagulase-positive staphylococci) was the rabbit plasma tube test, using 0.5 mL rabbit plasma (Benex Limited, Dublin, Ireland). A CAMP test was carried out to confirm *Streptococcus agalactiae* among the presumptive streptococci. *Streptococcus uberis* was confirmed via the lack of visible colonies on the bile-esculin agar but with gradual hydrolysis of the esculin around the inoculation line. Gram-negative bacteria were subcultured onto MacConkey agar N2, and the red colonies with a precipitation zone were confirmed as *Escherichia coli*. Other pathogens were classified to the genus level (coagulase-negative staphylococci or *Staphylococcus* spp., *Streptococcus* spp., *Enterococcus* spp., and *Corynebacterium* spp.).

According to the NMC guidelines [35] for the significance of colony numbers isolated in bacterial growth culture, either pure or mixed with other colony types (based on a 0.01 mL quarter milk sample streaked on blood agar), the degree of confidence in diagnosing an infection was expressed using five categories: no growth (0), no significant growth (1), questionably significant growth (2), probably significant growth (3), and highly significant growth (4). The single-colony growth of any organism was considered relevant if it was pure growth [34].

The number of colony-forming units (cfu) of every pathogen per 1 mL of the udder quarter milk was determined semi-quantitatively in four categories: 100 < 500, 500 < 1000, 1000 < 5000, and ≥5000 cfu/mL. This scale was adapted from a recent study on *Corynebacteria* [36].

### 2.4. Basic Diagnostic Interpretation of the MaP and MiP at the Udder’s Quarter Level

The number of mastitis pathogen colonies isolated from milk samples during laboratory testing was an important criterion. According to the NMC guidelines, all pathogen growth for the significance categories from 1 to 4 were combined and interpreted as the status for real a IMI. The joined category was compared against category 0 to evaluate subclinical intramammary infections with major and minor udder pathogens as a cause of an increased SCC in milk. An IMI was defined as a major pathogen (MaP) if *Staphylococcus aureus*, *Streptococcus agalactiae, Streptococcus dysgalactiae*, *Streptococcus uberis*, esculin-positive streptococci, *Escherichia coli*, *Klebsiella* spp., other coliforms, *Enterococcus* spp., *Trueperella pyogenes*, *Pasteurella* spp., or *Pseudomonas* spp. were identified. Growth of coagulase-negative staphylococci or *Corynebacterium bovis* were considered minor pathogens (MiPs) [24,26]. If only one species of mastitis pathogen was found in the milk sample, the quarter was classified as a single-pathogen-infected quarter. When an udder’s quarter was found positive for both MaPs and MiPs, it was evaluated as affected by an MaP in the summary classification (Table 2). The continuous presence of a pathogen was defined as the continued presence of the same pathogen in the same quarter of the udder at the first and second sampling times. The appearance of the pathogen was defined as the detection of bacteria in milk at the second sampling time but not at the first sampling time. The episodic presence of MaPs was defined as the detection of an MaP only at the first or second milk sampling time but at the other milk sampling time an MiP was isolated or no pathogen was found. An episodic MiP infection was defined as detecting an MiP at the first or second sampling time of the investigation but at the other sampling time the presence of another MiP or no pathogen was found (Table 2).

### 2.5. Aggregated Diagnostic Interpretation of the MaPs and MiPs at the Cow’s Udder Level

Cows with no mastitis pathogens detected in their milk samples were classified as pathogen free. The cow was classified as a single-pathogen-infected cow (single-pathogen status) if only one mastitis pathogen was found in the milk samples from several udder quarters. If two or more pathogens were detected in the different udder quarters, the cow was classified as multiple-pathogen-infected (multiple-pathogen status). In addition, the pathogen status was evaluated separately at the first and second milk sampling times; cows could maintain the same status or change it between the samplings.

### 2.6. Statistical Analysis and Two-Level Mixed-Effects Modeling

A two-level mixed-effects logistic regression modeling was performed in the software Stata (StataCorp LP, 4905 Lakeway Drive, College Station, TX, USA, version Stata BE 17.0 for Windows). The odds of the appearance of a mastitis pathogen in an udder quarter of a particular cow were interpreted using the independent variables of the cow’s lactation number, lactation length (standard lactation of 305 days; extended lactation of more than 305 days in milk), fat, protein, lactose, urea in milk, and a linear score for the SCC (LS_SCC_) at the first and second sampling times. As previously described, the SCC data were converted into LS_SCC_ data using Equation (1) [37,38].

LS_SCC_ = ln(SCC × 10^−5^) × (ln2)^−1^ + 3 (1)

Using univariable logistic regression [39], all independent variables were first screened for the direct association with the probability of the minor mastitis pathogen’s emergence in the quarter of the udder at the second milk sampling compared with the first milk sampling. Only those that were significant at a liberal alpha level of 0.25 were further included in the multivariable modeling. The full model and nested reduced model were compared using the likelihood ratio test to evaluate the final model’s necessity for each variable. Not included independent variables were put, one by one, back into the final two-level mixed-effects logistic regression model to demonstrate the *p*-value in the presence of a set of main predictors (LS_SCC_ at the first sampling, extended lactation of more than 305 days in milk, and their interaction). The null hypotheses were rejected if the *p*-value was below the significance level of 0.05.

## 3. Results

### 3.1. Major and Minor Mastitis Pathogens at the Cow Udder Quarter Level

A set of primary data consisted of the results obtained from repeated udder-quarter-level bacteriological testing (the presence of MaPs and MiPs), chemical testing (fat, protein, lactose, urea), and cytological testing (somatic cell count) in 45 cows selected from the two herds. The following MaPs were identified in this study: coagulase-positive staphylococci, esculin-positive streptococci, and *Enterococcus* spp.; the MiPs identified were coagulase-negative staphylococci and *Corynebacterium* spp. However, it was possible for some quarters to have mixed infections and sometimes switch from one pathogen at the first milk sampling to another pathogen at the second milk sampling.

In total, 176 quarters in 45 dairy cows (41 with four functional quarters and 4 with three functioning quarters) were analyzed for the presence of MaPs and the MiPs (Table 3). In general, the distribution of the pathogen groups differed between the case and control cows (*p* < 0.05). The most pronounced differences were observed in situations with the episodic presence of MaPs (11/1) or no pathogen presence (15/25). There were similar sizes for the case and control subgroups (37/37) regarding the continuous presence of an MiP.

**Figure 1:**
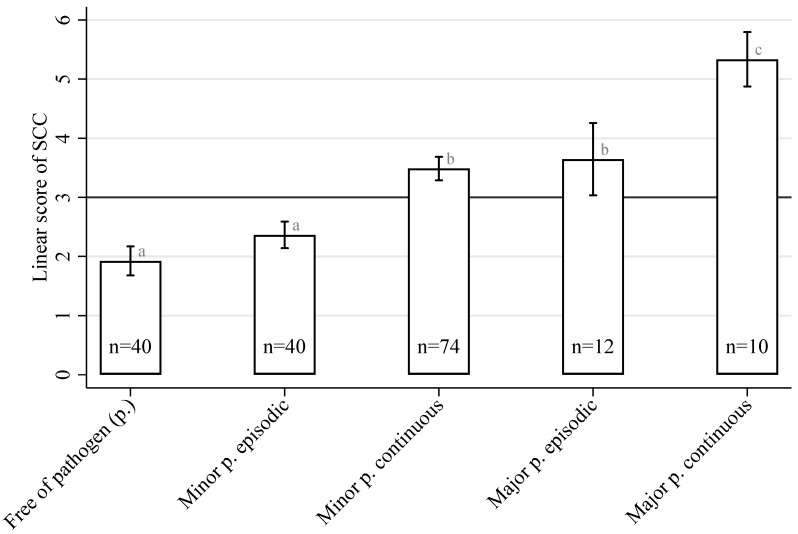
The mean linear score for SCCs in milk from a cow’s udder quarters depends on the pathogen (p.) isolation permanence (free/episodic/continuous) and pathogen groups (minor p./major p.). Bars with different superscripts (a–c) differ significantly from each other (*p* < 0.05).

The distribution of the major and minor pathogens in the cow udder quarters at two sampling times with 10- to 14-day intervals was closely related to the results of the quarter-level SCCs. Figure 1 illustrates the linear score for the SCC depending on the mastitis pathogen group and its permanence in the mammary gland. The lowest LS_SCC_ values (not exceeding 3 log_2_ units or 100,000 cells/mL) were found in milk from udder quarters with no pathogen presence or in cases of the episodic presence of an MiP. A higher (*p* < 0.05) mean value for the LS_SCC_ (but not exceeding 4 log_2_ units or 200,000 cells/mL) was detected during the continuous presence of an MiP. The same was true during the episodic presence of an MaP. However, a higher mean value for the LS_SCC_ of more than 4 log_2_ units or 200,000 cells/mL could not be excluded. The highest LS_SCC_, which exceeds 5 log_2_ units or 400,000 cells/mL, was found when the presence of an MaP was confirmed in both quarter milk samples.

### 3.2. Major and Minor Mastitis Pathogens at the Cow’s Udder Level

At the udder level, a single mastitis pathogen in the udder was found in 24.4% (11/45) of all cows at the first milk sampling time and in 35.6% (16/45) at the second milk sampling time (Table 4). Multiple pathogens were identified in 64.5% of cases (29/45) at the first sampling time and 62.2% of cases (28/45) at the second sampling time. The proportion of cows for which no mastitis pathogen was yielded was 11.1% (5/45) during the first sampling time and 2.2% (1/45) during the second sampling time. The first and second sampling times had similar proportions for single-, multiple-, or free-of-pathogen cows (*p* > 0.05). The disposition of mastitis pathogens across quarters in the cow’s udder differed for each bacterial group. Coagulase-positive staphylococci and *Enterococcus* spp. were generally detected in only one or two quarters out of four. Coagulase-negative staphylococci and esculin-positive streptococci were found more frequently in one or two quarters but much less often in three quarters. *Corynebacterium bovis* were more often detected in milk from one or two quarters. However, it could be present in milk from three or all four mammary glands of a single cow.

An appearance (a detection of bacteria in milk at the second time of sampling but not detected at the first sampling time) of the mastitis pathogens in quarters of the udder is shown in Table 5. Coagulase-positive staphylococci appeared in two cows at the second time of milk sampling compared with the first time. Although the appearance of coagulase-positive staphylococci in those cows concerned only one quarter, both cows corresponded to the multiple pathogen status as they still had another pathogen in the remaining three quarters. Notably, the percentage of cows without the appearance of any pathogen was 51.1% (23/45). The appearance of a combination of two different pathogens was only in one cow, and the cows most often showed the appearance of only one new pathogen.

### 3.3. Modelling of the Factors Associated with the Appearance of Mastitis Pathogens

The factors directly associated with the probability of the minor mastitis pathogen’s emergence in a quarter of the udder at the second milk sampling time compared with the first milk sampling time are presented in the table (Table 6). In terms of cow-level parameters, extended lactation (OR ± SE = 1.91 ± 1.04; *p* < 0.25) and the cow-level LS_SCC_ in the current month (OR ± SE = 0.80 ± 0.14; *p* < 0.25) were associated with the odds of a minor mastitis pathogen appearance at the second sampling compared with the first milk sampling. In terms of the quarter-level parameters, core variables for the appearance of the minor mastitis pathogens were the LS_SCC_ (OR ± SE = 0.71 ± 0.11; *p* < 0.05) and milk lactose (OR ± SE = 2.30 ± 1.28; *p* < 0.25) at the first milk sampling time. All these variables were further included in the building process of the multivariable model.

Multivariable modeling (Table 7) showed that the presence of MiPs at the second milk sampling compared with the first sampling was more commonly observed in the quarters with initially lower SCCs. In the framework of modeling, the clustering of the udder quarters of each cow in one whole was detected as significant (*p* = 0.031), i.e., the probability of the appearance of mastitis pathogens in the quarters (Level 1) of a particular cow (Level 2) was moderately correlated with the quarters of the same cow’s udder (intraclass correlation coefficient ICC = 0.27 ± 0.16). According to the model, if the number of somatic cells in milk was one log unit lower, the possibility of a minor mastitis pathogen’s appearance increased on average 1.8 times (1/OR = 1/0.56; *p* < 0.05) for any standard-lactation cow. However, a significant interaction between the LS_SCC_ at the first sampling and the number of lactation days suggested that this was not true for cows in the extended lactation phase. For any cow with extended lactation of more than 305 days in milk, the possibility of the minor mastitis pathogen’s appearance increased on average only 1.06 times if the number of somatic cells in milk decreased by one log unit (1/OR = 1/(0.56 × 1.69); *p* < 0.05).

## 4. Discussion

The current study demonstrated the effects of subclinical infections by udder pathogens grouped as major (MaP) and minor (MiP) on the somatic cell count (SCC) in the milk of cows milked using DeLaval milking robots. To discriminate between an episodic presence and a continuous presence for an udder pathogen in milk, a comparison was applied to the results from the bacteriological testing of two milk samples obtained with an interval of 10 to 14 days. Such a longitudinal approach to studying the continuity of the pathogens using several milk samples from the udder quarters has been suggested more often than it has been implemented in the design of even large-scale studies [20]. The current study bacteriologically compared two milk sampling times to better evaluate the udder pathogen status. Compared with a single sampling, triplicate samples provided the most precise diagnostic results. However, such an approach ensured only a modest gain in specificity and little or no gain in the sensitivity of udder pathogen detection. According to some authors, the selection of the sampling method needs to be tailored to the goals of the study or clinical investigation [10].

From a practical point of view, the sensing of the udder pathogens in conventional milking was performed via the bacteriological testing of the milk samples; this was only performed after the observation of clinical mastitis signs during milking or signals from subclinical mastitis sensors or a high SCC for the cow in the monthly milk recording data. In the future, a newly developed biosensor will perhaps be available for direct on-site identification and quantification of mastitis-causing pathogens in milk, as this is already demonstrated for some pathogens by analyzing milk samples from cows that are suffering from acute clinical mastitis [40]. Since the implementation of MRs, the early indirect sensing of IMIs through the detection of deviations in the milk associated with bacterial udder pathogens and an inflammatory reaction has been fully transferred from human action to sensor measurements [14]. As described by Kamphuis et al. (2013) [7], the mastitis detection system should be an effective on-farm tool for managing the quality of the produced milk; in other words, ensuring acceptably low SCCs in produced milk via the timely detection of cows with clinical mastitis and identifying cows with high SCCs. However, only some MRs are equipped with sensors able to perform detection of SCCs in milk at the cow level and it is even less common for MRs to be able to do this at the udder quarter level. Commonly, in the standard version of an MR, sensors for electrical conductivity measurements are installed to allow immediate recognition of cows with clinical mastitis [41]. Hypothetically, the same sensors could be implemented to track and follow up subclinical intramammary infections with udder pathogens—the current study aimed to investigate the sensing of bacterial pathogen emergence using the MDi. In general, to understand the dynamics of the SCC, it was worth using sensors to trace the permanence of the pathogen and whether it belongs to the group of minor or major pathogens, such as continuous MiP infections and episodic or continuous MaP infections.

Conventional microbiological testing is extensively accepted as a background tool for diagnosing intramammary infections (IMIs) [11,20]. In the current study, the quarter foremilk bacteriological testing results indicated continuous IMIs with MaPs in 5.7% and MiPs in 42.0% of all udder quarters. The prevalence of continuous IMIs with MiPs was comparable with other authors’ studies [20], where they found a prevalence for non-aureus staphylococci at the levels of 50.6 and 20.6% for quarters with SCCs above or below 200,000 cells/mL, respectively. Additionally, the presence of other MiPs, such as *Corynebacterium* spp., in milk was often high [36]. According to published data [42], udder infections with MiPs decrease a cow’s lactation yield on average by 5.7% in the case of coagulase-negative staphylococci infection and by 7.4% in *C. bovis* infection and this justified the bacteriological examination of quarter milk samples even in cases where only MiPs were detected. However, other authors’ research has shown that the pathogen’s diagnostic sensitivity using the cow’s whole milk sample for bacteriological examination was lower than 80% (61.2 or 59.8% if only one quarter was affected by *S. uberis* or coagulase-negative staphylococci, respectively) [43]. However, the bacteriological testing of the cow’s whole milk would be acceptable before drying off to decide the type of dry cow treatment with or without antibiotics.

This study showed the presence of *C. bovis* at the first and second sampling times as well as the emergence of *C. bovis* occurring most often in one quarter or two quarters; however, it was possible that it was in three quarters at the same time (see Table 4 and Table 5). Such a localization for *C. bovis* highlighted the importance of corynebacterial prevalence in milking farms with an MR. In line with some other research, if *Corynebacterium* spp. was bacteriologically detected in the milk, even the quarters with an SCC lower than 100,000 cells/mL should be considered latently infected [36].

Other research [25] has shown that 47% of milk samples with SCCs higher than 500,000 cells/mL and a negative initial bacteriological test result could be recognized as positive on repeated testing with more sensitive methods such as post-freezing bacteriology and polymerase chain reaction. In turn, among cases where only the growth of an MiP, such as *Corynebacterium* spp. or coagulase-negative staphylococci, was detected, molecular diagnostics also revealed the presence of an MaP in 35 and 25% of samples, respectively. This hidden MaP was often identified as *E. coli*, *S. uberis*, *S. dysgalactiae,* or *Trueperella pyogenes*. This fact could also be applied to the interpretation of our research results because many of the udder quarters (15 out of 74) with the continuous presence of an MiP in milk had SCCs higher than 500,000 cells/mL and in several of these quarters an MaP was not found, perhaps due to the detection method used.

The appearance of pathogens or a new infection was mainly observed in one or two mammary glands of cows during the second sampling compared with the first sampling. At the same time, only in 2.2% of cows (1/45) did a new infection occur in three quarters; the simultaneous appearance of a new pathogen in all four quarters of the udder was not detected. A wide array of herd- and cow-level defense mechanisms of course played a role against IMIs, such as teat/teat canal characteristics, cellular immunity, breed, genotype, age, stage of lactation, SCC, milk yield, energy balance, nutrition, and viral infections, as well as amount of exposure to mastitis pathogens due to hygiene, existing/previous intramammary infections, and the microbiota of the teat skin [31]. The spread of pathogens among cows was affected by many factors. Not all of them were analyzed in the current scientific research project due to the main focus being on tracing the udder pathogens using milk quality sensors built into the DeLaval MR.

In large-scale studies, there is an opportunity to study each species of MiP separately, allowing for the observation of many differences. However, MiPs impacted the SCCs between bacteriologically negative quarters and udder quarters infected with MaPs [20,44]. Therefore, in the present study, the effects of subclinical infections by udder pathogens were compared between the groups of MaPs and MiPs, each split into the episodic or continuous presence of the pathogen in the milk. A limitation of the current study was the low number of cows with an episodic presence of an MaP; therefore, at the second milk sampling, the factors associated with the appearance of an MaP were not evaluated by the logistic regression method (it was detected in only two udder quarters, Table 5). The quarters with the continuous presence of an MaP contributed most to the SCC increases. For such cows/udder quarters, an early mastitis treatment was indicated without delay. In case of the necessity for repeated treatment, replacement of the cow with a new animal might be considered because, along with the continuous presence of a pathogen, the risk of biofilm formation increases in the mammary gland. Therefore, chronic mastitis could develop [45].

The current study showed the increasing probability of finding the appearance of a new MiP in the udder quarters of a cow with an SCC below 200,000 cells/mL. However, an extended lactation of longer than 305 days was not a significant factor in the appearance of an MiP. This aligns with other authors’ findings that IMIs by coagulase-negative staphylococci may be observed at any lactation phase and that days in milk were not a significant predictor for an IMI. However, the spectrum of coagulase-negative staphylococci species may change with an increasing number of days in milk [20,44].

## 5. Conclusions

The mean SCC in the milk of an udder’s quarter depends on the pathogen’s group and the continuity of the pathogen’s presence in the milk. The impact of major pathogens on the SCC is relevant for both episodic and continuous presences. However, minor pathogens impact the SCC only when they continuously reside in the quarter.

According to the multivariable regression model, minor mastitis pathogens for new infections were more common in the quarters with initially lower SCCs.

The novel finding of this study was that by using the milk quality sensor for the sensing of subclinical IMIs, there was an indication for the successful detection of episodic MaP infections. However, the sensing of continuous subclinical IMIs was not possible in the current study and still needs to be investigated.

## Figures and Tables

**Table 1:** The number of cows in the case and control groups according to the lactation number and days in milk.

Parity	Lactation Phases ^1^								Total
	Early		Middle		Late		Extended		
	Case	Control	Case	Control	Case	Control	Case	Control	
1st	0	2	1	1	3	3	3	4	17
2nd	0	0	1	1	2	2	2	2	10
≥3rd	0	1	1	1	5	5	3	2	18
Total cows	0	3	3	3	10	10	8	8	45

^1^ Lactation phases by number of days in milk: early phase 10–100, middle phase 101–200, late phase 201–305, and extended lactation > 305 days.

**Table 2:** Diagnostic interpretation (grouping) of mastitis pathogens at the cow’s udder quarter level.

The Permanence of the Pathogen’s Presence	The Group of Pathogens		Summary Classification of the Pathogen’s Group
	At the 1st Sampling	At the 2nd Sampling ^1^	
Continuous	MaP	MaP	Major pathogen continuous
	MiP	MiP	Minor pathogen continuous
Episodic	MaP	None	Major pathogen episodic
	None	MaP	
	MaP	MiP	
	MiP	None	Minor pathogen episodic
	None	MiP	
	MiP	MiP ^2^	
No presence	None	None	Free of pathogen

^1^ The second sampling of the milk was conducted 10 to 14 days after the first sampling time; ^2^ A different minor bacterial pathogen was found at the second sampling time.

**Table 3:** Distribution of the major pathogens (MaPs) and minor pathogens (MiPs) in the udder quarters of the cows in the case and control groups during repeated sampling.

Summary Classification of the Pathogen Group	The Number of Cow Udder Quarters (%)	The Ratio between Case ^1^/Control ^2^ Groups
Continuous MaP	10 (5.7%)	4/6
Continuous MiP	74 (42.0%)	37/37
Episodic MaP	12 (6.8%)	11/1
Episodic MiP	40 (22.7%)	14/26
Free of pathogens	40 (22.7%)	15/25
Total	176 (100%)	81/95
The difference case/control	…	*p* = 0.008

^1^ Cows with MDi ≥ 1.4; ^2^ Cows with MDi < 1.4.

**Table 4:** The disposition of mastitis pathogens across quarters in the cow’s udder as a single- or multiple-pathogen infection.

Diagnosis Level	The Disposition of Pathogens in the Cow’s Udder	Coagulase-Positive Staphylococci	Coagulase-Negative Staphylococci	Esculin-Positive Streptococci	*Enterococcus* spp.	*Corynebacterium* spp.	Number of Cows Number of Cows, % (Case ^1^ + Control ^2^)		
							With the Pathogen in Any of the Quarters	Free of Pathogen in All Quarters	In Total
At the first sampling time ^3^									
Cow udder Level ^4^	Single pathogen	-	3	1	-	7	11 24.4% (5 + 6)	5 11.1% (1 + 4)	45 100% (21 + 24)
	in 1 quarter	-	1	-	-	3			
	in 2 quarters	-	2	-	-	2			
	in 3 quarters	-	-	1	-	-			
	in 4 quarters	-	-	-	-	2			
	Multiple pathogen	[5] ^5^	[28] ^5^	[8] ^5^	[2] ^5^	[24] ^5^	29 64.5% (15 + 14)		
	in 1 quarter	5	24	4	1	10			
	in 2 quarters	-	2	2	1	7			
	in 3 quarters	-	2	2	-	5			
	in 4 quarters	-	-	-	-	2			
At the second sampling time ^3^									
Cow udder level	Single pathogen	-	3	-	-	13	16 35.6% (6 + 10)	1 2.2% (1 + 0)	45 100% (21 + 24)
	in 1 quarter	-	1	-	-	3			
	in 2 quarters	-	2	-	-	4			
	in 3 quarters	-	-	-	-	2			
	in 4 quarters	-	-	-	-	4			
	Multiple pathogen	[4] ^5^	[24] ^5^	[6] ^5^	[1] ^5^	[27] ^5^	28 62.2% (14 + 14)		
	in 1 quarter	3	13	3	1	11			
	in 2 quarters	1	7	1	-	9			
	in 3 quarters	-	4	2	-	4			
	in 4 quarters	-	-	-	-	3			

^1^ Cows with MDi ≥ 1.4; ^2^ Cows with MDi < 1.4; ^3^ All numbers in the Table refer to the number of cows; ^4^ Each cow was evaluated by the number of pathogen-affected quarters, the number of pathogens (“single pathogen” means only one pathogen, “multiple pathogen” means more than one pathogen in the same cow) at the first and second sampling respectively; ^5^ These numbers do not sum up horizontally because in the cases of “multiple pathogen” each cow was included in several columns in this table.

**Table 5:** The appearance of the mastitis pathogens in quarter milk samples from the individual cows and the persistence of single- and multiple-pathogen status.

Mastitis Pathogen in Milk	Number of Cows ^1^				Total (%)
	Keeping ^2^ the Pathogen Status		Changing ^3^ the Pathogen Status		
	Single	Multiple	Single	Multiple	
Coagulase-positive staphylococci	-	2 2/0/0/0	-	-	2
Coagulase-negative staphylococci	-	5 4/1/0/0	2 1/1/0/0	-	7
*Corynebacterium* spp.	-	4 2/2/0/0	5 0/5/0/0	3 2/0/1/0	12
Combination of pathogens (coagulase-negative staphylococci and *Corynebacterium* spp.)	-	-	-	1 ^4^ 0/1/0/0	1
The sum of cows with the appearance of any pathogen or pathogen combination	-	11	7	4	22 (48.9)
Number of cows without the appearance of any pathogen	5	12	4	2	23 (51.1)
Total, cows	5	23	11	6	45 (100.0)

^1^ In the second line are shown the changes in the pathogen in one/two/three/four quarters; ^2^ The same single-pathogen or multiple-pathogen status was detected at the first and second sampling times; ^3^ A change in the single-pathogen or multiple-pathogen status of a cow was detected between the first and second sampling times; ^4^ Each pathogen in a separate quarter.

**Table 6:** Initial screening of the different factors to evaluate their direct association with the appearance of a minor pathogen in the sample of cow’s milk from udder quarters (n = 164 udder quarters).

Factors ^1^	Mean	SE ^2^	Min	Max	OR ^3^ ± SE	Chi-Squared Statistic	*p*-Value
Udder quarter-level LS_SCC_ ^4^ at the first sampling (log_2_ units)	2.93	0.16	0.00	8.14	0.71 ± 0.11	4.63	0.032
Udder quarter-level lactose at first sampling (%)	4.53	0.04	2.47	5.26	2.30 ± 1.28	2.25	0.133
Cow-level LS_SCC_ ^4^ in the current month (log_2_ units)	3.19	0.13	0.36	6.63	0.80 ± 0.14	1.53	0.216
Standard lactation of ≤305 (days)	212.24	14.08	47	293	Reference category		
Extended lactation of >305 (days)	390.33	19.68	313	537	1.91 ± 1.04	1.42	0.233

^1^ The factors shown in this table are sorted by ascending order of P-values up to *p* < 0.25 in univariable mixed-effects logistic regression; ^2^ Standard error of the mean; ^3^ Odds ratio from univariable mixed-effects logistic regression; ^4^ A linear score for the SCC.

**Table 7:** Positive or negative effects of multivariable predictors in the logistic regression model on the appearance of minor mastitis pathogens in the cow udder quarters (n = 164).

Predictors	Mean ± SE ^1^	OR ^2^ ± SE	*p*-Value	95% CI ^3^	Effect
A ^4^: LS_SCC_ (log_2_ units) ^5^	2.93 ± 0.16	0.56 ± 0.10	0.001	0.40 … 0.80	Negative
Standard lactation of ≤305 (days)	212.24 ± 14.08	Reference category			
B ^4^: Extended lactation of >305 (days)	390.33 ± 19.68	0.65 ± 0.45	0.529	0.16 … 2.53	Not significant
Interaction: A × B	…	1.69 ± 0.0.38	0.020	1.09 … 2.63	Positive
Constant	…	0.63 ± 0.25	0.234	0.29 … 1.35	…

^1^ Standard error of the mean; ^2^ Odds ratio from multivariable mixed-effects logistic regression; ^3^ The 95% confidence interval for the OR; ^4^ Abbreviature to indicate the interaction between the predictors; ^5^ A linear score for udder quarter SCC at the first sampling time.

## Data Availability

The data that support all results of the present study are available on request from the corresponding author.
